# How can we best help this patient? Exploring mental health therapists’ reflections on medication-free care for patients with psychosis in Norway

**DOI:** 10.1186/s13033-022-00529-8

**Published:** 2022-04-04

**Authors:** Christine Henriksen Oedegaard, Ana Lorena Ruano, Anne Blindheim, Marius Veseth, Brynjulf Stige, Larry Davidson, Ingunn Marie Stadskleiv Engebretsen

**Affiliations:** 1 Centre for International Health, Department of Global Public Health and Primary Care, University of Bergen, Bergen, Norway; 2 Division of Psychiatry, Haukeland University Hospital, Bergen, Norway; 3 Department of Psychiatry, Yale Medical School, 319 Peck Street, Building 1, New Haven, CT 06513 USA; 4 The Grieg Academy, University of Bergen, Bergen, Norway; 5 Department of Clinical Psychology, University of Bergen, Bergen, Norway

**Keywords:** Norway, Health care delivery, Psychosis, Policy implementation, Street-level bureaucrats, Medication-free treatment

## Abstract

**Background:**

Since 2015, Norwegian Regional Health Authorities have followed new government policy and gradually implemented medication-free services for patients with psychosis. The aim of this qualitative study was to explore the tension between policy and practice, and how health care workers in Bergen reflect on their role in implementing medication-free treatment.

**Methods:**

We performed three focus group discussions including 17 therapists working within medication free services, asking about their experiences with this new treatment program. We used Systematic Text Condensation for data analysis. The findings were discussed using Michael Lipsky’s theoretical framework on the role public health workers play in policy implementation.

**Findings:**

Following Norway’s new policy was challenging for the therapists in our study, particularly balancing a patient’s needs with treatment guidelines, the legal framework and available resources. Therapists had an overarching wish to help patients through cooperation and therapeutic alliance, but their alliance was sometimes fragile, and the therapists worried about patients’ conditions worsening.

**Conclusions:**

Democratization of treatment choices, with the aim of empowering patients in mental health care, challenges the level of professional discretion given that patients and therapists might have conflicting goals. Balancing the desire to help, professional responsibility, the perceived lack of resources, and certain patient choices created conditions that can leave therapists feeling disempowered in and alienated from their work.

*Trial registration*: N/A.

**Supplementary Information:**

The online version contains supplementary material available at 10.1186/s13033-022-00529-8.

## Background

Over recent decades, there has been a shift from a paternalistic role of the physician acting in the assumed best interest of the patient toward an increased emphasis on the will of the patient [1]. In this paradigmatic shift, the focus on decisional capacity and patients being seen as right holders is increasingly important in mental health care. Human rights activists criticize the use of coercive treatment and disempowerment of the patient and emphasize the individual freedom to choose treatment [2]. However, patients’ freedom to choose treatment within mental health care can be demanding for health workers, because they have a professional obligation to protect the patient and the community from harm and must take these perspectives on treatment and care into account [3, 4].

Health care in Norway is government funded. As in most health care systems, the delivery of care is subject to the prioritization of available resources, including the type of medication provided, to available psychosocial treatment options. Available resources depend on both the Regional Health Authorities’ priorities and government policy. The Minister of Health in Norway established a policy in 2014, stating that mental health care should increase more than health care for physical illness in terms of both the use of resources and level of treatment activity within each region. The success of this policy is debated, as resources provided for somatic (physical) health care still increase more than for mental health care [5]. Resources within mental health care have mainly been reallocated from inpatient to outpatient treatment [6], and there has been limited increase in the total level of resources. Further, it is generally understood that patients with psychosis or schizophrenia are a resource-demanding patient group. In 2018, a report from the Norwegian Directorate of Health showed that 10% of the adult patients in mental health care used 76.7% of the resources for that sector. The report also states that patients with schizophrenia are overrepresented within this group [7].

Schizophrenia spectrum disorders represent severe mental illnesses that imply a high global disease burden and disability [8], and are often treated with pharmacotherapy [9]. In 2011, service user organizations came together to lobby for medication-free services within the Norwegian mental health care system [10]. This service requirement also emerged from the debate on the effectiveness and adverse effects of antipsychotic medication (AP) used as part of the treatment for severe mental illness [11–32]. The debate on the implementation of medication-free treatment was polarized, with professionals arguing against it, pointing at research showing that medication works, and a lack of scientific support for the new guidelines [33]. The Norwegian government agreed with user organizations that medication-free services should be a priority, and in 2015, the Norwegian Regional Health Authorities began allocating resources and introducing these services for patients with psychosis, within the constraint of the law defining responsible treatment [34]. The law provides constraints implying that all patients 18 and above, who are able to give an informed consent and are not subject to coercive treatment, can choose medication-free treatment. If the patient is sentenced by court to coercive treatment, or the patient is considered to lack ability to give an informed consent, and/or is considered dangerous, patients are not allowed to discontinue their medication if their therapist consider medication necessary. Medication-free treatment aims to support patients wishing to discontinue their medication in a safe environment.

Medication-free treatment in Bergen is provided in district psychiatric clinics, generating more psychosocial treatment options for people suffering from psychosis. The treatment options consist of recovery-oriented services such as peer support, supported employment, and illness self-management [35]. It is a recovery oriented reform of mental care focusing on the patient’s human right to make decisions regarding their treatment [36–39]. The staff consists of health care workers with different professional backgrounds, including, but not limited to, psychiatrists, psychologists, music therapists, nurses, social educators, physiotherapists, occupational therapists, and social workers. In this study, when we refer to “therapists”, we include all mental health care staff. Providing patients with medication-free treatment options is considered by both policy makers and user organizations to be a step toward recovery-oriented care. This includes increased patient autonomy and democratization of the patient–therapist relationship, focusing on shared decision-making, which is defined as a form of patient–therapist communication in which both parties are acknowledged to bring expertise to the process and work in partnership to make a decision [40].

Frontline health workers in this study translated new laws, guidelines, and treatment options into practice in everyday health care delivery as part of the medication-free treatment project. Their role as mediators and interpreters of policy helped shape its implementation. To our knowledge, there is only one previously published paper focusing on psychiatrists’ perspective in the implementation of medication-free treatment [41], and no study specifically focusing on the therapists’ role as front line workers translating new policies into treatment practice in this regard. To better understand how these therapists actively shaped the way public policy on medication-free services was implemented, we used Lipsky’s theoretical framework regarding street-level bureaucrats (SLB) [42]. He defines street-level bureaucracies as agencies whose workers, the SLB, interact with, and have wide discretion over, the dispensation of benefits or the allocations of public sanctions. Mental health workers provide benefits and sanctions to their patients and have the authority and agency to make and carry out discretionary decisions with relative autonomy from management.

This provides a useful theoretical framework for interpreting the role mental health care providers play in policy implementation. Our aim was to explore how mental health care workers in Bergen dealt with, and reflected on, the challenges in implementing this new policy regarding medication-free treatment.

## Method

### Study context

This study was performed in Bergen, a city in western Norway, where the implementation of medication-free services was launched as a project in September 2017. That project aimed to standardize this type of care, to ensure that all district psychiatric centers offered the same psychosocial treatment options.

All adult patients within the Bergen catchment area who are suffering from psychosis and are not subject to coercive treatment can choose medication-free treatment. Patients who are subject to coercive treatment can choose to participate in the various psychosocial treatment options but are not free to discontinue medication without consent from the psychiatrist or the court. Choices regarding treatment alternatives, like individual music therapy, are to some extent limited by availability, but most options are available within a reasonable time frame. Treatment is voluntary, with no mandatory components. Medication-free treatment should follow the guidelines otherwise provided for treating psychosis, allowing a careful discontinuation of the medication, and adding more psychosocial treatment options to support the patient in this process. This means the patient aims at discontinuing medication, but this is a process allowing dosage reduction and increase following patient wishes and symptom load.

In Norway, music therapy is highly recommended in the guidelines for psychotic disorders, referring to high evidence rating supporting it as treatment. Music therapists are mental health workers with a high degree of independence in the performance and choices regarding the treatment of their patients. At times, they may end up being the only therapist seeing a patient on a regular basis, and they can choose to increase or decrease the frequency of therapy, and ask for more or less support from the other mental health care team members regarding patients’ needs. Thus, we believe music therapists meet the criteria as SLB, and were included in a separate focus group discussion.

### Theoretical framework

People come to street-level bureaucracies, such as health care facilities, as individuals with unique personalities, experiences and circumstances in their lives. In the encounters with these bureaucracies, they are transformed into clients through a social process in the effort of making them fit into standardized definitions of units consigned to specific bureaucratic slots. Lipsky calls this process the social construction of the client [42]. In the context of medication-free services, patients are clients who enter potentially conflict-based relationships with SLB because they may clash over objectives and because both parties have different levels and amounts of resources with which to negotiate paths forward. Clients seek services and benefits, and SLB seek control over the process of providing them. In the context of new treatment options emphasizing patient choice, the social construction of the client is aiming toward a more horizontal and less hierarchical structure.

According to Lipsky, SLB often must navigate the tension between what is demanded from them professionally, by both patients and management, and what they are able to provide within the given conditions. Large caseloads, ambiguous agency goals, and inadequate resources strain mental health providers, while the demand for services tends to increase with the supply. Hence, tasks may often be hampered by resource constraints. If the tension becomes too demanding, SLB may experience feelings of alienation from their work, because they experience a loss of control over situations they are expected to handle with authority. This may lead to feeling dissonance, and when this dissonance between objectives and capabilities is too great, SLB may develop coping mechanisms to shield them from the implications of the gap. Such coping mechanisms includes disclaiming their responsibility towards their patients, consciously or subconsciously, and emphasizing the division between work and private life. This alienation from their work leads to dissatisfaction with the job, in turn affecting commitment to patients and their agencies.

### Study design

This was a qualitative study performed as part of a doctoral project exploring patient and therapist perspectives on medication-free treatment of psychosis. Qualitative methods are research strategies to describe, analyze and interpret experiences of people as they encounter, engage and live through situations, providing diversity and nuances for the scientific knowledge pool. We chose focus group discussions to explore purposely selected therapists’ experiences, attitudes and perspectives with the implementation of medication-free treatment for people with psychosis.

### Recruitment

To recruit participants for the two first focus groups we approached the directors of the three public district psychiatric centers within the Bergen area, who supported the initiative. The directors provided us with a list of names of key personnel involved in the medication-free treatment, all working with patients suffering from psychosis within a district psychiatric center in Bergen. We sent an e-mail with an invitation to participate to the therapists on the list. All the invited participants (n = 12) agreed to participate and provided informed consent prior to study participation. One person could not participate for personal reasons on the day of the discussion.

Music therapists were invited in a separate focus group discussion, where we also included music therapists from three adjacent private clinics that worked with medication-free treatment in collaboration with the public clinics. The music therapists were all recruited through the POLYFON Knowledge Cluster for Music Therapy, where both the public and the private clinics were members. All agreed to participate (n = 6) and provided informed consent prior to participation.

### Participants

We held three focus group discussions in autumn 2017 and spring 2018 with health personnel, as shown in Table 1.

**Table 1:** Focus group participants

Focus group 1, December 2017 District Psychiatric Clinic Psychiatrists and psychologists	Focus group 2, June 2018 District Psychiatric Clinic Bachelor-level education	Focus group 3, June 2018 University of Bergen Music therapists
Moderator: CHO Secretary: MV	Moderator: CHO Secretary: MV	Moderator: CHO Secretary: BS
P* 1: Male 60–70 Psychiatrist	P1: Male, 40–50 Mental health nurse	P1: Male, 30–40 Master of music therapy
P2: Male, 40–50 Psychologist	P2: Female, 30–40 Physiotherapist	P2: Male, 50–60 PhD in music therapy
P3: Female, 50–60 Psychologist	P3: Female, 50–60 Occupational therapist	P3: Female, 20–30 Master of music therapy
P4: Female, 40–50 Psychiatrist	P4: Female, 40–50 Social educator	P4: Male, 20–30 Master of music therapy
P5: Female, 40–50 Psychologist	P5: Male, 40–50 Occupational therapist	P5: Male, 30–40 Master of music therapy
P6: Female, 40–50 Psychiatrist		P6: Male, 30–40 Master of music therapy

^*^P participant

The participants in the first focus group were psychiatrists and psychologists, two men and four women, age range from about 40 to about 70. The participants in the second group were one nurse, one physiotherapist, two occupational therapists, and one social educator, two men and three women, age range from about 30 to about 60 years old. The participants in the third group were all music therapists, one woman and five men, age range from about 25 to about 50.

### Data collection

The first two focus groups lasted for 60 min, while the last had additional questions specifically concerning music therapy and lasted for 90 min. Participants were clustered according to their professional background to create a familiar and safe environment for the free sharing of experiences. We followed Malterud’s recommendations for organizing the focus groups to determine number of participants, length, and moderator/secretary roles, and also for using a thematic questionnaire, by asking for concrete incidents and stories [43]. The questionnaires are available as supplementary material. The first author audiotaped and transcribed the focus group discussions.

The qualitative design prompted verbal interaction and elaborate discussions between health personnel, who shared their experiences with the medication-free treatment program. We asked the participants to describe their experiences discussing treatment choices with patients, and their ways to approach shared decision making. Further, we asked the participants to share their worst experiences treating patients, concerning drop-out, and/or worsening. The focus group topic guide was open for both positive as well as negative consequences of the implementation of medication free therapy. Finally, we asked how they experienced the level of available resources and support from the management.

### Data analysis

For analysis purposes, we used Systematic Text Condensation (STC) [44], a method inspired by Giorgi’s psychological phenomenological analysis [45]. This is a thematic, cross-case strategy suited for exploratory analysis, consisting of five steps: identifying preliminary themes; identifying meaning units in this case concerning therapists’ challenges and concerns regarding medication-free treatment; sorting the meaning units into code groups; abstracting condensates from code groups and sub-groups; and finally, generating synthesized accounts of the main concerns for the therapists. The main author and two co-authors read the transcripts, and each found between five and eight preliminary themes relevant across all three focus groups. Further, they prioritized five of the most substantial themes. The main author and one co-author organized the meaning units, identifying those potentially related to the previously chosen themes. We elaborated on the names and keywords of the code groups during coding to develop understanding. The main author wrote the text condensates, reducing the content of the meaning units into a concentrated text or short story describing the main views expressed in the focus group discussions regarding the specific meaning units within the chosen themes, retaining the participants’ terminology as much as possible. Meaning units that could not be incorporated in the condensate were set aside, with some reorganized into other themes, and some excluded due to lack of relevance. Each of the condensates was discussed with two co-authors. The analytical process resulted in three themes: managing available resources; the role of the therapist; and treatment practices and experiences. To finalize the analysis process, the condensate was rewritten into the results section, returning iteratively to the original transcribed text to check the validity of each meaning unit in the condensate. In this process, the main author translated the text into English, validated by two co-authors.

To remain close to the voice of the users, experts-by-experience were co-researchers throughout the entire research process from design to dissemination of this study, including the analyzing process. The findings were discussed using Lipsky’s theory presented above.

### Ethical clearance

The Regional Ethics Committee for Medical Health Research (REK sør-øst 2017/736) defined this study as health service research and hence according to the Norwegian Health Research legislation, the study was approved by the local data protection officer for Health Bergen in July 2017 (2017/8692).

## Results

Analysis as described in the method section revealed three main themes; managing available resources; negotiating the role of the therapist; and treatment practices and experiences. The following are condensates based on the coded meaning units from the three focus group discussions.

### Managing resources in the mental health services

The participants described patients with psychosis in general as requiring significant resources, and several expressed an impression that medication-free patients were often among those requiring more resources than patients who used medication. They worried about relapses, and the process of recovering after relapses was described as time consuming for patients suffering from psychosis, with months of inpatient treatment. When patients were believed to be worsening, the focus was to increase the support in every way possible, if the mental health care team could get into position to treat. This was described as a challenge, as patients worsening often refused help before they were acutely admitted, and the worry was this would be non-voluntary. During this type of admissions, the treatment was described to secure the patients’ life and health in the acute department. For patients experiencing periods of worsening, the focus was on stabilizing inpatient treatment with sleep, rest, and medication. Inpatient departments in the district psychiatric clinics did not have a systematic medication-free treatment regimen, although they offered cognitive behavioral therapy (CBT), nutrition management, and physiotherapy. The pressure on available beds often led to patients being discharged as soon as possible, often as soon as they were well enough to utilize treatment methods other than stabilizing measures.

The participants considered it important to provide medication-free patients with extended support and close follow-up to avoid worsening and possibly acute admissions, but described situations when it was difficult to agree on replacing medication with other treatment options:

Psychologist: *When the patients have insight and cooperate using treatment options other than medication, then it works fine, you make it work. However, if there is no insight, and they do not want to or are unable to utilize other treatment options, then it gets difficult*.

Moderator: *What do you do then?*

Psychologist: *Then you search in the available “menu”, really, and see if there is anything that could work, kind of meet the needs, depending on the treatments offered.*

All patients were thought to benefit from all or several of the treatment measures implemented, but the capacity of the therapy, including group size and available therapists, was limited. Regular discussions related to prioritizing medication-free patients over patients using medication took place:

Music therapist: *And then, it is like, ok, but should they be prioritized more for music therapy, or should everyone get the same. And I think considering our workload, do we really have the resources to provide more for those choosing a medication-free treatment course? Not really. And then it is a challenge considering how music therapy also is a resource, because patients come and go, there are waiting lists, and then the waiting list is not all rigid, right, so, if someone arrives and we see that, this one has to get it (music therapy), then this person gets ahead of others.*

Therapists tried to motivate medication-free patients to stay connected with the clinic and in treatment by pushing them to attend some form of therapy regularly. They believed that this pressure to attend led to less motivated patients in therapy, and subsequently to frustration for both patient and therapist, especially when patients did not attend therapy sessions:

Music therapist: *For patients actively choosing medication-free treatment, it is important to consider if it is responsible treatment, which is what the doctor keeps in the back of their mind. I have thought about it a lot in those situations—you have to replace it [medication] with something. So, that depends on an agreement; now you have to use music therapy, or other options, right? And then this is when you see they stop coming. (…) How long should I wait, and let them come now and then, sometimes a month between sessions. Then, it is not so responsible. Then, you have to do something.*

Therapists could not use resources on treatment measures that after a given time had no effect on the symptoms or functioning of the patient, and they often had to consider how long they should wait before giving the opportunity to the next patient on the waiting list. This contrasted with the understanding that this patient group needed to spend time in new settings before feeling safe, and that the treatment alliance needed to develop over time for the treatment process to succeed.

Patients with psychosis in general were said to often need close follow-up over time outside the hospital, in facilitated school or workplaces, practical aid, and social activation, and medication-free patients were sometimes described to be very resource demanding in this regard. A problem with discharging patients was the increased need for relatives to provide support, because public health services did not provide enough:

Mental health nurse: *The question is how long you can impose on family or others to keep such a close contact, because public health care does not offer that much in everyday life.*

The therapists also described how many patients had small or no family or networks and relied on the health personnel taking care of them. Participants also felt they could not discharge patients if the patients had nowhere to go, so they avoided to discharge and stretched the guidelines to do this. Several mentioned a lack of adapted housing offered by the municipality as the worst problem when discharging patients:

Moderator: *Have you been out checking on the living conditions for your patients?*

Psychologist: *Some are homeless.*

Psychiatrist: *Yes, they live in the inpatient clinic, right, the clinic is supposed to be used by patients in need of acute admission [but] those who need acute admission are hindered, because patients in need of adapted housing cannot be discharged—they would perish*.

Patients living in the clinic are more resource demanding, and medication was described to often be a stabilizing factor allowing the patients to be discharged and be able to make use of the housing they were offered.

### Negotiating the role of the therapist between guidelines and patient relationships

All therapists in our study assessed the patients’ stories and their previous medical history to adjust the treatment according to their specific needs, both regarding pharmacological and non-pharmacological treatment. They focused on providing the patients with thorough information about recommended treatment and the options available to them to make an informed choice. The main goal was to help the patients by providing descriptions of treatment practice:

Mental health nurse: *But it has been tried with several approaches, and of course, here medication is a part of the treatment, but it has never been the idea that medication should be the only treatment. Our main focus has been cognitive therapy, that for that matter is medication-free treatment. But then, several struggles with utilization of this in a period when it is all chaos, right? (…) Because we know that some really has good effect of the medication, and others don’t.*

Moderator: *Yes. And what do you do with those who do not have any effect of the medication?*

Mental health Nurse: *Yes, what helps, in a way, right? That is always the question, how can we help this patient in the best way possible, with or without medication*.

The psychiatrists in our study, responsible for the medication, reported that medication-free treatment was something they had always practiced, and they cooperated with patients who chose to reduce or discontinue medication:

Psychiatrist*: It has never been a problem to work towards a pause or discontinuation of medication with patients who have insight, who relate to the illness, who can warn us about worsening, who do not have problems becoming dangerous; this was not a problem even before this [medication-free treatment] was initiated.*

The psychiatrists emphasized that when medication was prescribed, it was generally together with psychotherapy, and not as the only treatment. The aim was to give accurate information about benefits and adverse effects and find the correct medicine and dosage for each patient. Additionally, the psychiatrists were preoccupied with identifying and helping those who experienced little or no effects of the medication. The psychiatrists emphasized their flexible attitude towards medication as they thought the patients, and the user organizations working to implement medication-free treatment, often misunderstood this.

All the health care workers in our study, regardless of professional background, emphasized the importance of keeping a good relationship with the patient, cooperating as much as possible:

Occupational therapist: *Because they should see us as a part of a health-care system wanting to help them. We should not be pushy, we should not be there just because they happen to be referred to us, but we should actually want to help them, show empathy, and be available.*

The role held by different health care workers in mental health services changes in accordance with the phases of the patient’s illness and how their symptoms fluctuate. In this study, therapists described how assessing a patient’s insight could be complicated and difficult, along with their ability to give informed consent, and the potential danger they posed to themselves or others. Official treatment guidelines required appropriate and professional treatment approaches:

Psychologist: *It can be quite tricky with the young patients, who may have had several episodes, and then they want to discontinue the medication, and in a way, you can discuss it, but the guidelines are quite clear, having several episodes in a row is not an indication for quitting medication right away, at least.*

The therapists concluded that following guidelines for when to use medication could be an obstacle to obtaining, and maintaining, a good relationship with the patients.

### Collaborating about treatment strategies and choices

The therapists reported that they were sometimes surprised by patients coping well without medication, thereby admitting being unable to predict possible outcome of discontinuation for all patients. They believed including several perspectives on treatment in team-based decision-making was important, leaning on each other’s competence and varying connection and alliance with the patient. One music therapist described how other therapists used him when the patient was interested in music, so they could reach a position where other treatments could be provided. The music therapist was able to build a therapeutic relationship with the patient before other health care team members could, and this alliance could then be used to add other treatment types as the patient felt safer. This could avoid the patient falling out of treatment. He emphasized how it was important to be a team, and not to be the only responsible health care worker, especially when patients appeared unstable:

Music therapist: *But then it is so important not be alone. (…) It’s easy for me to say “I do not feel competent to consider this. I need somebody else to engage.” I can tell them what I have observed, but if I feel somebody else needs to get involved, they do. That gives a sense of security.*

The participants emphasized the importance of spending time figuring out what were the real priorities for each patient and discussing pros and cons for each treatment decision that was made. The best option was to reach an agreement in cooperation with the patient on a long-term treatment plan, even if they were sometimes impatient to get well. This became increasingly complicated if the patients did not want to use medication but managed poorly without it:

Psychologist: *But then he becomes so sick he is no longer capable of taking care of himself. Then it’s not possible to cooperate without medication, because he would just disappear, he wouldn’t utilize the other treatment options.*

In situations where the patient was lacking insight, or when, for some reason, they did not want treatment or contact with the health system, the fear of the patient worsening was challenging for all the therapists. They described how it was difficult to see patients on the street, living under terrible housing conditions, or listening to relatives talking about upsetting outcomes. However, the intention to respect and accept patient choices was clear, although it included a sense of resignation related to their wish to help:

Psychiatrist:* … and it is visible in the streets that some people make bad choices, and I believe we must learn to think that, ok, we do our best, but in the end the patient decides, unless they are dangerous.*

## Discussion

The therapists in this study described treatment strategies when coping with managing resources and situations where needs were difficult to meet. Patients who chose medication-free treatment were reported to need extended support and other treatment measures with close follow-up to succeed. The therapists communicated how their role was based on an overarching wish to help patients, which was difficult to balance with conformance to guidelines, laws, and available resources in treatment practice. Shared decision-making and spending time considering patient preferences was perceived to be important in the treatment process. The alliance was sometimes fragile, and periods of patients worsening were worrisome.

### Discretion and prioritizing

Health care services are paradoxical in the sense that care is delivered by people to people, requiring human interaction and caring, but also delivered through a bureaucracy, which invokes a model of detachment and equal treatment under conditions of resource limitations and constraints. The delivery of street-level policy through bureaucracy depends on health care workers’ abilities to embrace this paradox [42]. One example of resource deficiency from our study was the lack of adapted housing within the municipality. This kept patients admitted longer than needed. Specialist mental health supported housing is considered key to a graduated level of care from institutionalized to independent living in the community [46]. The participants in this study wanted to secure the best solution to the problem of housing for their patients. The problem of discharging was solved by keeping patients in care longer than the guidelines suggested was necessary. This occurred because mental health therapists feared their patients would perish without necessary support once they were outside the institution. This stretching of their allowed discretion was possible because SLB, such as these mental healthcare therapists, are able to use and interpret rules and constraints that are externally imposed upon them to achieve their preferred ends [42]. Other examples of resource deficiency were waiting lists to attend music therapy, or simply patients having needs that the mental care system could not meet. Often, this meant that the relatives were more burdened with taking care of their own than the therapists would consider sustainable in the long term. Research has shown that family members caring for relatives with schizophrenia experience a high level of objective and subjective burdens [47]. Additionally, scholarship suggests a higher degree of relapse and mortality when patients discontinue anti-psychotic medication [48]. Hence, the therapists worried this burden would increase when patients chose to discontinue medication.

Patients who chose medication-free treatment in this study were considered to need extended support and treatment measures, and it was believed that success required close follow-up. The implementation of medication-free services in Bergen has enlarged the available treatment options in district psychiatric clinics. The therapists indicated that they discussed problems with prioritizing medication-free patients over other patients. Careful consideration of individual needs was perceived to be the best way to decide whether or not the patient needed medication and was the main tool for prioritizing treatment measures. This process was supposed to be mainly controlled by patient wishes and perceived needs, rather than the therapists’ discretion.

Psychosocial support measures are recommended, and are already validated as efficient in the recovery process for patients suffering from severe mental illness, including schizophrenia. Evidence based measures are available and constitutes a so-called “menu” from which the therapists can make informed choices and present to the patient [49]. The process of shared decision-making corresponds with recommended approaches to enhance the relationship with and the recovery process of the patient [40]. At the same time, it is shown how discontinuing anti-psychotic medication might have a negative impact regarding relapses, defined as increased hospitalization, and mortality. Psychosocial measures are resource demanding, and will inevitably meet requirements of cost-effectiveness in a health care system with limited funding. These requirements will be managed by therapists, as SLB, trying to balance both the implementation of a more resource-demanding treatment, held together with the increased risk of patients worsening and hence needing more resources in their follow-up. Prioritization is a part of the difficult task balancing human care with the demand for equal treatment within limited resources. Medication-free treatment seems to require more human resources, as well as additional human and other resources for close follow-up if a patient is worsening. This stands in conflict with cost–benefit demands of the Norwegian mental healthcare system and its guidelines on the use of resources. Efficiency in resource use is an organization-centered goal, and requires that SLB prioritize in their role as gatekeepers. This may affect elements of care for their patients.

### Ambiguity and complexity

SLB typically have jobs with conflicting and ambiguous goals [42]. Within mental health care, this might be even more evident when patients (i) claim they are not sick, and hence do not need treatment, (ii) are not satisfied with the treatment they are offered and therefore do not want their help, and/or (iii) are subjected to coercive treatment. All these aspects were raised by the therapists in our study, although coercive treatment only as something to avoid. In health care systems, the defined goal is to provide the best possible treatment and care for all patients, a client-centered goal. A person experiencing a physical illness, like cancer or heart disease, is most likely to seek professional help. However, for mental health care, it is paradoxical that higher symptom load would predict a lower likelihood for that person to seek help [50]. Hence, the client-centered goal might be challenging to achieve when patients do not want the help they are offered.

The ambiguity of the task also surfaces in the relationship the therapists have with their patients. Clients of most bureaucratic systems, including health care systems, are non-voluntary; street-level bureaucracies provide essential services that citizens cannot obtain elsewhere. Hence, patients in mental health care may be non-voluntary in more than one sense; both as a client of a bureaucratic system providing an essential service unobtainable elsewhere, but also as a person suffering from an illness where their help seeking behavior is largely affected by the symptom load as described in the paradox above.

The therapists in this study, although expressing an overarching aim to help patients, felt ambiguity when balancing improved patient influence and their ability to provide essential services. When patients understand the concept of help differently than the therapist, their role was to resolve these conflicting perceptions, and to provide treatment perceived as acceptable and useful to the patient within the available resources. Providing patients with thorough information about treatment choices, including medication, was important. SLB in our study interpreted intensified information sharing as a way to fulfill the need and demand for shared decision-making [51].

Critics of medical authority in mental health services have described the Norwegian system as one that uses patriarchal ways of communication rather than patient-centered decision-making [52, 53]. This study indicates the participants intended to promote a democratic mode of decision-making, which is consistent with a study that explored psychiatrists’ attitudes toward shared decision-making [54]. The therapists in this study emphasized the importance of spending time carefully considering pros and cons together with the patient to avoid hasty decision-making, and to accept patient choices even when they worried about the outcome.

In this context of emphasizing choice and shared decision-making, the social construction of the client–SLB relationship aims toward a more horizontal structure. However, shared decision-making is at the core of the conflict between the two possibly diverging perceptions of the kind of help needed and can be difficult to negotiate. Society and management expect professional discretion and responsibility in decisions affecting health care delivery. As such, the options offered must be within the scope of available resources, laws, and guidelines. Because there are resource and time constraints, health personnel should be provided with a range of relevant treatment options, from which they can build an appropriate treatment menu for each patient, process the information and produce an appropriate response to patient needs [42]. In this study, this is described as the treatment ‘menu’ presented to the patient. This is a way to reduce the complexity of treating mental illness to a manageable level of choices for the health personnel, but runs the risk of reducing the influence of the patient if the patient is presenting needs outside of the available “menu”. The structure of the simplification or routines in presenting a “menu” of choices creates a low-level decision-making environment, where the frame is politically determined, and the presentation of choices is at the discretion of the therapist. In this sense, the SLB in this study shape medication-free policy, allocating available goods and services, ideally, but not necessarily, based on mutual consent of patient needs.

### Concerns within the therapeutic alliance

In mental health care provision, the patient’s level of symptoms and illness largely dictate which law and guidelines are at play. Many of the therapists in our study expressed how they worried about their patients worsening, because of the possible implications for the patient, and for the change in relationship and responsibility for the therapist. One participant described how a patient became so sick he could no longer take care of himself. This implies the therapist must take on a different role, where shared decision-making is no longer perceived as useful and forced treatment has to be considered. In this phase of psychosis, when a patient’s paranoid tendencies and withdrawal from interactions with others may lead them to avoid therapy, the therapists in our study became concerned about their patients’ safety and sought ways to keep them in treatment using their agency as SLB. Additionally, patients may have delusions about what might work to reduce their symptoms and ask for measures outside treatment guidelines and resources. However, denying a patient’s request can be highly uncomfortable [55], and requires specific skills from the therapist. This becomes especially complicated when patient safety is conditioned by the fragile alliance between health personnel and the patient [56]. Although the therapeutic alliance is suggested to be a key component in successful mental health care delivery [57, 58], some studies have found no evidence that alliance predicts the outcome of complex psychiatric treatment for patients with psychosis [59]. Nevertheless, this provides a backdrop for how health care workers understand and interpret their role, particularly when therapists in this study worried about patients worsening and quitting all therapy. When the relationship with one therapist was endangered by turning down patient requests, use of other health care team members was described as a way to remain in a position to treat the patient. Communicating limitations in patient choices while maintaining a good relationship with the patient is a challenging dilemma in mental health care delivery.

When patients chose to withdraw from treatment, even if the therapist perceived their symptoms as worsening, the therapists in their roles as SLB in our study felt they had to respect and accept the patients’ choice. Acceptance of a patient’s choice that may lead to deterioration in their somatic, mental, social, or physical quality of life may be interpreted as alienation from therapists’ key function, which is to safeguard all their patients. This is similar to the findings from a recent study of sources and features of moral distress experienced by acute psychiatric nurses. The feeling of being squeezed between ideals of good care and a harsher clinical reality caused bad conscience, feelings of inadequacy, and emotional numbness. The study concluded with how moral distress may lead to reduced quality of care with nurses distancing and disconnecting themselves from their patients and their inner selves [60]. The implementation of medication-free treatment was by many psychiatrists in particular not regarded as based in a scientific view on professional and good care of patients with severe mental illness [41]. This debate is well known in the society [61]. Hence, when there is an experienced dissonance between objectives (cure/help the patient) and capabilities (personal and resource/system-related limitations), workers develop mechanisms such as alienation to shield them from the implications of the gap between expectations and accomplishment [42]. In our study, the implementation of the medication-free treatment might have added to the range of treatments and the emphasis on shared decision making for the patients, but it may also be resource demanding and a source of worry and distress for the therapists.

### Reflexivity, strengths, and limitations

This article focuses on how therapists experience the challenges regarding the implementation of medication-free treatment, rather than the opportunities provided by the same policy change. This limitation of the study scope gives more space to explore these challenges. The down side is the lack of focus on the positive aspects given in the data, including the possibilities provided by such a change of policy. Additionally, we need to include the experiences of other stakeholders, such as relatives and patients in addition to already existing studies on this topic [62, 63].

The researcher’s background and position will inevitably influence the outcome of a study by affecting choice of topic, choice of methods, and framing of findings and conclusions. Contemporary theory of knowledge disputes the belief of the neutral observer [64]. Reflexivity has been a guiding principle that has given our interdisciplinary team of coauthors room to discuss and reflect on all aspects of the study, from design to dissemination. Importantly, experts-by-experience were co-researchers throughout the entire research process, which we believe has strengthened the trustworthiness of the study, providing feedback from the most important voices, the users of the health care system.

The scope of the data collection was limited to one context in Norway, and we cannot assume that our findings are similar in other implementation settings. However, the use of theoretical framework structured our interpretation and presentation to focus common themes in policy implementation in health care workers’ roles, such as democratization of the therapist–patient relationship, level of discretion, and management of resources.

Choosing Lipsky’s theoretical framework has helped clarify how health workers are affected by policy implementation and how they navigate the ways in which they decided to put it into practice in their everyday work. Nevertheless, this framework was developed in the 1960s and 1970s in the United States. The differences in cultures, health systems and contexts could have affected the interpretation. Additionally, this framework may not have been sufficient in addressing some important aspects, such as different professional roles and the relationship between workers and management [65]. Professional roles influence the level of discretion, the level of freedom granted and cooperation between workers and management, and it would have been interesting to explore how different health workers perceived the influence of their professional background and hierarchical situation on their experience of medication-free treatment implementation. This was, however, not the topic of the study.

At the time of the focus groups discussions, the implementation of medication-free services was just starting. This might have affected the extent to which health care workers had experience with and felt familiar with patients choosing to discontinue their medication. On the other hand, this may have led to policy implementation and changes in practice being fresh in the mind of the participants.

For future research, we suggest looking into one key factor in decisions regarding treatment, which is how therapists consider patients’ capacity for giving an informed consent.

## Conclusion

Health personnel in this study experienced all the ambiguity and complexity that the work of SLB entails because the democratization of treatment choices in mental health care challenges the level of professional discretion. While the aim is to empower patients, this restricts the SLB ability to make decisions and can be perceived as lowering their agency. The implementation of a recovery-oriented medication-free treatment in daily practice in this study resulted in conflicting goals. Balancing the wish to help and professional responsibility with perceived lack of resources and troublesome patient choices created the conditions that may lead to therapists feeling disempowered in and alienated from their work.

## Supplementary Information

**Additional file 1.** Topic guide for focus group discussions 1 and 2.

**Additional file 2.** Topic guide for focus group discussion 3.

## Data Availability

The transcribed focus groups are not publicly available for confidentiality reasons, but anonymized Norwegian transcripts can be made available from the corresponding author on reasonable request.

## Abbreviations

AP: Antipsychotic medication (AP)
SLB: Street-level bureaucrats (SLB)
STC: Systematic Text Condensation (STC)
CBT: Cognitive behavioral therapy (CBT
