# Supplementary material for: How can we best help this patient? Exploring mental health therapists’ reflections on medication-free care for patients with psychosis in Norway
Source: Int J Ment Health Syst. 2022 Apr 4;16:19. doi: 10.1186/s13033-022-00529-8 (PMC8978409; doi:10.1186/s13033-022-00529-8)
Supplement: Supplementary file 1 — Additional file 1. Topic guide for focus group discussions 1 and 2. [file 13033_2022_529_MOESM1_ESM.docx]

# Topic guide for focus group discussions

Focus: The therapists' experiences with the medication-free treatment offer at the various DPSs in Bergen.

Process-oriented topic guide, what works well, what do they find difficult? Why?

Focus on concrete stories!

## Background - narrative

You are invited here today to share your experiences with the fact that patients with psychotic disorders can now choose treatment without antipsychotics.

The temperature in discussions in the media has at times been high both in terms of what is responsible treatment, and how an informed consent should be assessed, but especially in relation to patients' experience of coercion and lack of autonomy in their own treatment, and thus in their own lives. We want to focus on the specific challenges you as therapists face every day, especially in relation to the implementation of this treatment offer. The topic of the discussion will thus be therapists' specific experiences with patients who want medication-free treatment, either as part of the drug-free treatment offer at Kronstad, or during "treatment as usual". What is challenging about this? What works well?

## Shared decision-making

What kind of experiences do you have from discussions with patients about the choice of treatment?

Do you feel that the patient's thoughts about choices and needs regarding treatment are adequately taken into account? Why, why not?

What kind of help do you feel patients need the most?

Which part of the treatment do you trust the most? Why?

## The needs of therapists

What is the worst thing you experience as therapists in relation to patients? Do you want to share some experiences?

-suicide

-drop-out

-worsening

-cooperation with other therapists

-cooperation with other agencies

-cooperation with relatives

## Experience of available resources

Is there a form of treatment that you experience as in demand, but which you can not offer?

How do you envision that (the medication-free) treatment offer would be in a world where everything worked out, and you could choose to put together the treatment offer exactly as you wanted?

## Experience of and need of support

What is the most important thing for you as a therapist to get help with when you encounter problems in the treatment of a patient?

Do you as a therapist experience that you get the help and support you need from the employer to be able to optimize the treatment of the patients?
