# Supplementary material for: How can we best help this patient? Exploring mental health therapists’ reflections on medication-free care for patients with psychosis in Norway
Source: Int J Ment Health Syst. 2022 Apr 4;16:19. doi: 10.1186/s13033-022-00529-8 (PMC8978409; doi:10.1186/s13033-022-00529-8)
Supplement: Supplementary file 2 — Additional file 2. Topic guide for focus group discussion 3. [file 13033_2022_529_MOESM2_ESM.docx]

Interview guide - focus group discussion with music therapists

Focus: Music therapists' experiences with the medication-free treatment offer at the various DPSs in Bergen.

Process-oriented interview guide, what works well, what do they find difficult? Why?

Focus on concrete stories!

Background - narrative

You are invited here today to share your experiences with the fact that patients with psychotic disorders can choose treatment without antipsychotics, and music therapy is part of this treatment offer.

The main focus of the discussion today will be:

1. Stories you hear from users who experience that music therapy works or does not work?

2. Own experiences of when and how music therapy works, and of situations and processes where music therapy does NOT work.

3. When do you experience that it does not work, and what do you do then? (how they handle it, how they collaborate with the user about it, whether / how they seek support in the professional environment, etc.).

4. And to what extent and in what way do you experience that users become involved in a shared decision-making process regarding their own treatment, can they freely choose music therapy if they wish? Are there any restrictions? To what extent can they choose the form of their own therapy? (choose to have it, choose content, choose to end, etc.)?

Further:

**The needs of therapists**

What is the worst thing you experience as therapists in relation to patients? Do you want to share some experiences?

**Experienced available resources**

Is there a form of treatment that you experience as in demand, but which you can not offer?

**Experience of and need of support**

Do you feel that you get the help and support you need from the employer to be able to optimize the treatment?
